# Satisfaction as a Mediator and Its Interaction With Adherence to Labor Analgesia Protocols: A Cross-Sectional Survey of Chinese Medical Personnel

**DOI:** 10.3389/fpubh.2022.899515

**Published:** 2022-06-28

**Authors:** Dong Lang, Chengxu Long, Shuna Lin, Yinghua Xie, Fangfei Chen, Rui Zhao, Chunping Liu, Shangfeng Tang

**Affiliations:** ^1^School of Medicine and Health Management, Tongji Medical College, Huazhong University of Science and Technology, Wuhan, China; ^2^Research Center for Rural Health Service, Key Research Institute of Humanities & Social Sciences of Hubei Provincial Department of Education, Wuhan, China; ^3^Department of Global Health & Social Medicine, King's College London, London, United Kingdom; ^4^China National Health Development Research Center, Beijing, China

**Keywords:** adherence, labor analgesia, personnel medical, maternal health, China

## Abstract

**Background:**

Although the Chinese promotion of labor analgesia began in 2018 to improve maternal health, high-quality medical care is difficult to provide to pregnant women when medical staff cannot implement standard labor analgesia procedures. This study aims to examine medical personnel's adherence to labor analgesia protocols and to explore the relationships among adherence, satisfaction, and other factors.

**Methods:**

The data were from a national cross-sectional dataset (*N* = 13,944) of the 2020 Chinese Labor Analgesia Pilot Evaluation Project. Mediating and moderating effects analyses were used to examine the role of satisfaction as a mediator between support measures and adherence.

**Results:**

There were differences in adherence between different types of medical personnel. Support measures and satisfaction had a positive association with adherence to labor analgesia protocols. Satisfaction had a significant mediating and moderating effect on the relationship between support measures and adherence to labor analgesia standards. Moderating effects of professional titles and attitudes were also observed.

**Conclusion:**

Primary health care policies worth considering include comprehensive incentives for medical institutions to improve the use of labor analgesia by medical personnel. It is also worth considering providing more training opportunities for the staff in anesthesiology departments.

## Introduction

Maternal health influences the quality of maternal life, the birth of healthy babies, and the stability of society; thus, it is a global concern (1). Health policies in many countries have made significant progress in improving maternal health (2, 3). The United Nations Sustainable Development Goals (SDGs) proposed accelerating the improvement of maternal health. Maternal health was also a focus of the Global Strategy for Women's, Children's, and Adolescent's Health 2016–2030 led by the WHO. However, in 2020, the global maternal mortality rate was 12/100,000, and the rate was 16.9/100,000 in China (4). Most maternal deaths in developing countries could be prevented if women received timely care during childbirth such as labor analgesia (5). The situation in developing countries is of great concern.

Developing countries have many problems threatening maternal health, while developed countries have successfully reduced maternal deaths by up to 80% through adequate funding, highly qualified personnel, and advanced technology (6). Labor analgesia technology has been shown to effectively promote maternal health (7). There is a disparity between developing and developed countries regarding the prevalence of labor analgesia. The rate of labor analgesia use in developed countries reached above 80% at the beginning of the twenty-first century (8–11). To improve maternal health, many developing countries are promoting labor analgesia (12, 13). However, only a few medical institutions offer adequate pain treatment during childbirth in China, and the rate of labor analgesia was only approximately 10% in 2017 (14). Labor analgesia has just begun to be used as an effective intervention to relieve labor pain in developing countries, and there are many issues that need to be addressed. To motivate further development of labor analgesia, the National Health Commission of China (NHCC) issued the Developing Labor Analgesia Pilot Work in 2018, and 913 hospitals were included in the first pilot in 2019.

A review of the available literature showed that standardized labor analgesia was of great significance for reducing the prevalence of cesarean section, improving the quality of life after labor (15, 16), and meet women's needs for painless labor (17). The committee of labor analgesia experts of the Chinese Medical Doctor Association compiled *the Clinical Standardized Management Path for Labor Analgesia in China*, which stated that an entire labor analgesia service should include promotion, professional operation, record keeping, and follow-up services (18). However, labor analgesia in China is still in the developing stage, and a large number of medical personnel are still learning basic skills. Irregularities may unavoidably occur during the implementation of labor analgesia. Consequently, determining medical personnel's adherence to standard labor analgesia plays an essential role in improving maternal health. In addition, the evidence suggests that women who chose labor analgesia are affected by the information provided by medical personnel and the quality of the labor analgesia (19–21). Adherence to labor analgesia protocols by medical personnel is essential for both maternal health and the well-being of society. Thus, this study aims to examine the factors related to the administration of labor analgesia, which offers insights to identify target groups, provides information for improving adherence to labor analgesia protocols, and provides implications for other developing countries facing similar issues.

## Literature Review

### Adherence to Labor Analgesia Protocols and Support Measures

Labor analgesia is crucial for pregnant women to live healthy life (7). Previous studies have identified implementing standardized labor analgesia has contributed to reducing the prevalence of cesarean section and improving pregnant women's physical and mental health (22). However, low-quality labor analgesia may cause serious complications (23); thus, it is important for medical personnel to adhere to analgesia measures. Adherence to labor analgesia protocols is also an important indicator in evaluating the progress and effectiveness of labor analgesia policies (24).

Since the promotion of labor analgesia in China, the influence of support measures on adherence to labor analgesia has been examined rarely. Lack of incentives and unavailability of equipment or personnel will influence labor analgesia adherence (25). Medical personnel often have negative attitudes to labor analgesia due to the scarcity of knowledge (26). It has been reported that incentives reduce the gap between medical personnel's knowledge and clinical practice with a large gain in efficiency (27). Added incentives for labor analgesia implementers could increase medical personnel's enthusiasm and improve the quality of service (28).

In addition, evidence has shown that one of the factors that affect labor analgesia in public hospitals is the lack of medical personnel and detailed charging items that is related to pain relief (29). Related arrangements for labor analgesia consist of four components—adequate personnel, adequate equipment, reasonable medical reimbursement, and charging items (18). Previous research has found that the better the arrangements are, the higher the rate and quality of labor analgesia (30). Based on the discussion above, this study is aimed to evaluate the relationship between support measures on labor analgesia.

### Satisfaction With the Implementation of Labor Analgesia

Support measures are probably to have positive effects on departmental collaboration satisfaction and consequently labor analgesia adherence, but the mechanism by which satisfaction relates to the relationship between labor analgesia adherence and support measures has not been specified in the literature. The existing evidence points to relevant perspectives in understanding this relationship. Existing evidence provides work satisfaction is closely related to the quality of medical services (31). Medical personnel with high work satisfaction are more likely to provide high-quality services. Meanwhile, work-related factors have a significant effect on work satisfaction (32). Labor analgesia can only be conducted with multidisciplinary cooperation (33). Specifically, anesthesiologists need to understand the whole process of labor and delivery, and obstetricians need to know the key points and operational techniques of labor analgesia, while midwives spend the longest time with women in labor (34). Highly intensive cooperation can result in timely and correct treatment to ensure maternal safety and guarantee the therapeutic effectiveness of maternal analgesia (35). Numerous studies have demonstrated the positive effects of adequate personnel and reasonable policies on the teamwork of medical personnel (36–38).

Professional development satisfaction exerts a powerful effect on improving the professional skill level of medical personnel (39). Since the NHCC implemented labor analgesia in 2018, medical institutions have implemented incentive measures that attract medical personnel by promoting them to professional positions. Adopting incentives was an excellent policy for those with lower-level professional titles (24, 40), but the incentive effect is limited to experts with senior professional titles. This is because seniors prefer to provide more professional guidance rather than practice labor analgesia (41). In addition, willingness is an indicator of satisfaction (42), and refers to whether medical personnel voluntarily participate in labor analgesia. Medical personnel are more willing to participate in labor analgesia when they are satisfied with the incentives and related arrangements (43). Meanwhile, medical personnel may be more inclined to use labor analgesia because there are fewer side effects and more effective pain treatment than previously reported (44).

**Figure 1:**
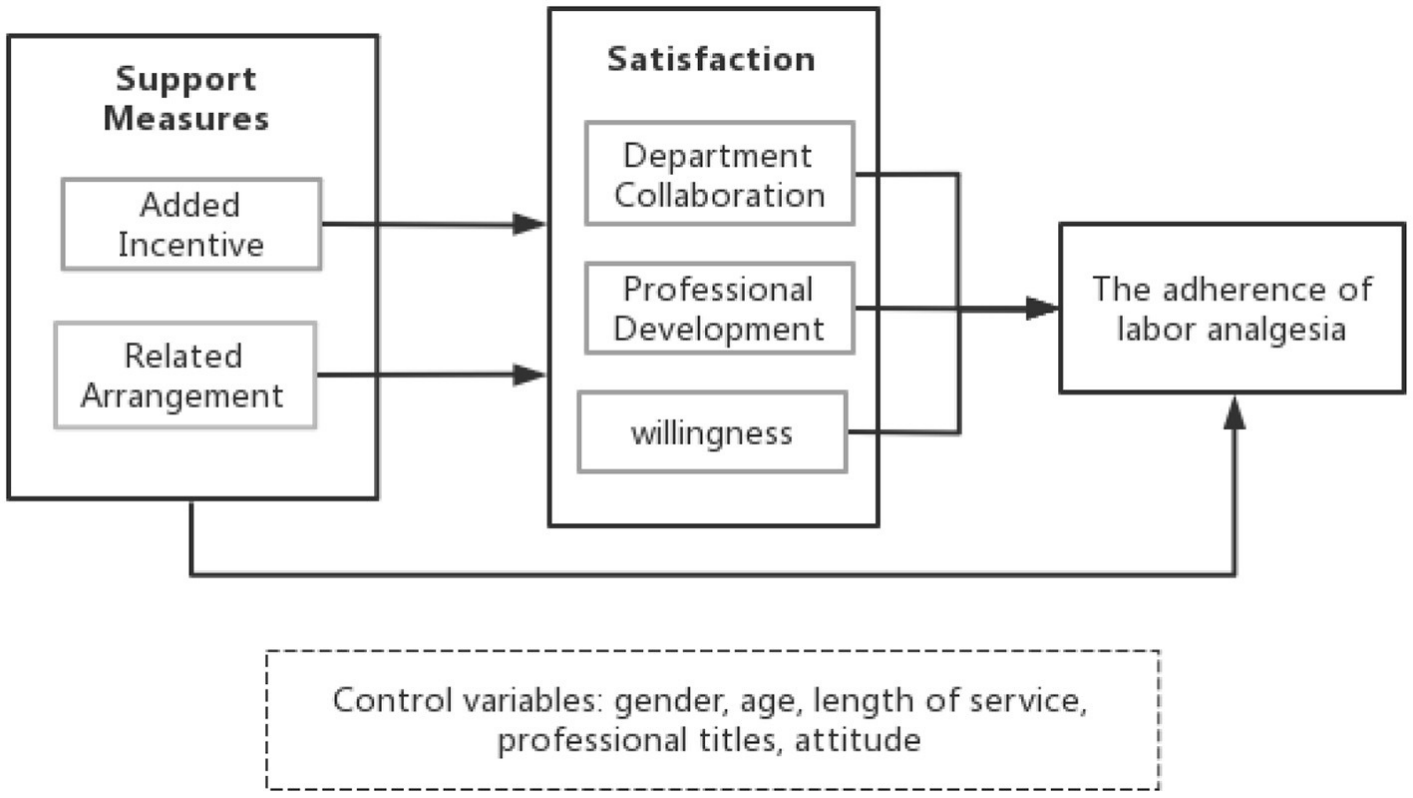
Conceptual framework.

So far, no studies have yet evaluated the role of satisfaction in mediating the relationship between support measures and labor analgesia adherence. Based on the discussion above, this study proposes a conceptual framework, as shown in Figure 1, in which support measures affect satisfaction with professional development and departmental collaboration and willingness affect adherence to labor analgesia. standards; that is, departmental collaboration satisfaction, professional development satisfaction, and willingness play mediating roles between support measures and adherence to labor analgesia protocols.

## Methods

### Data

The Chinese Association of Anesthesiologists surveyed to evaluate the progress and effectiveness of the Chinese national labor analgesia pilot programme with the help of provincial health administrations in 2020. All 913 pilot hospitals were invited to complete the related information in the system for the labor analgesia programme. The cluster sampling method was used for the investigation. All medical personnel in anesthesiology, obstetrics, and midwifery in each pilot hospital were invited to participate in the online questionnaire survey, which was developed by the team from Huazhong University of Science and Technology. Oral consent was obtained from all participants. A total of 13,944 questionnaires were sent out and 12,614 valid questionnaires were collected; the validity rate was 90.46%.

### Variables

Adherence to labor analgesia protocols was related to the support measures of the medical institution. As Table 1 shows, total adherence was selected as the dependent variable. Adherence comprised training for medical personnel and prenatal teaching for pregnant women and their family members. It also included four operative preparations: regular preoperative visits and records, adaptation training in the labor analgesia puncture position before labor, monitoring and recording throughout the administration of analgesia, and routine postoperative follow-up and recording after labor. The individual choices in the questionnaire were classified as Yes (1) and No (0). According to these six conditions, adherence to labor analgesia protocols was scored from 0 to 6 points.

**Table 1:** Dependent variables and assignments.

**Variables**	**Assignments**
**Dependent variable**	
Added incentives	1 = fairly unsatisfactory; 2 = unsatisfactory; 3 = general; 4 = satisfactory; 5 = fairly satisfactory
Related arrangement	Continuous variable (1–20)
**Satisfaction**	
Department collaboration	1 = fairly unsatisfactory; 2 = unsatisfactory; 3 = general; 4 = satisfactory; 5 = fairly satisfactory
Professional development	1 = fairly unsatisfactory; 2 = unsatisfactory; 3 = general; 4 = satisfactory; 5 = fairly satisfactory
Willingness	0 = no; 1 = yes
**Personal factors**	
Gender	1 = man; 2 = woman
Age	Continuous variable
Length of service	1 = 0–9 years; 2 = 10–19 years; 3 = 20–29 years; 4 = over 30 years
Professional titles	1 = primary and others; 2 = middle; 3 = vice-senior; 4 = Senior
Attitude	1 = strongly disagree; 2 = disagree; 3 = neutral; 4 = agree; 5 = strongly agree

Medical institutions' support measures were selected as the independent variable, including added incentives and related arrangements. Added incentives were graded on a scale of 1–5 to indicate “fairly unsatisfactory” to “fairly satisfactory”. According to the Clinical Standardized Management Path for Labor Analgesia, this study created a continuous variable, related arrangement, by adding up adequate staffing sufficient equipment, reasonable health insurance reimbursement, and charging entries, which was scored from 1 to 20 points. According to the availability of the relevant information in the database, the following variables were defined to explore the effect of the mediators and moderators: gender, age, length of service, department, occupation, professional title, and attitude. Among them, the length of service indicated the years that the medical staff had worked in the medical institutions. Attitudes referred to the medical personnel's beliefs about whether labor analgesia is valuable.

### Data Analysis

The data analysis consisted of analysis of variance, correlation analysis, mediating effect analysis, and moderating effect analysis. Analysis of variance was used to test the differences in adherence between different groups. Afterward, the Pearson correlation coefficient was used to estimate potential relations between the factors. Finally, mediating effect analysis was carried out to explore the relations between labor analgesia adherence and potential factors based on Baron's three-step method (45). The criteria for mediating effects were as follows: There was statistical significance between the independent variable and the dependent variable and between the independent and mediator variables. Then, the mediator in the regression model that included the independent variable and mediator was statistically significant (46). Similarly to the proposed methods (47), the moderating effect was tested by adding an interaction variable to the regression analysis to test both models. The first model contained potential variables and dependent variables (Model 1) and the second also included an interaction between the two variables (Model 2). And the interaction in Model 2 should be statistically significant when there are moderating effects. The data analysis was performed by SPSS 26.0. The results were regarded as significant when the *p*-value was < 0.05.

## Results

### Adherence to Labor Analgesia Protocols

**Figure 2:**
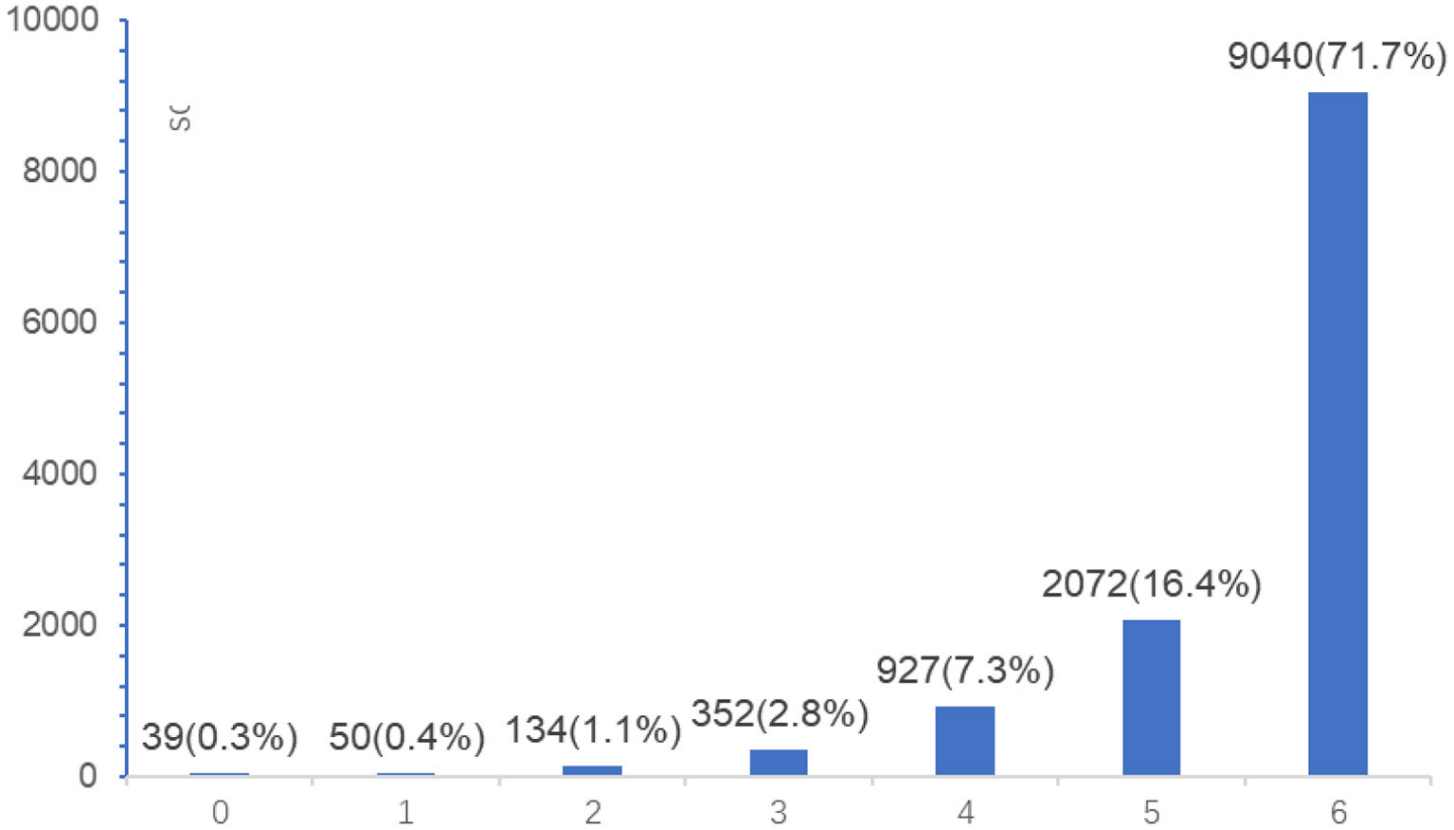
Adherence to labor analgesia protocols among medical personnel.

A total of 12,614 medical personnel participated in this survey. As shown in Figure 2, 71.7% of the medical personnel had the highest possible scores for adherence, and medical personnel with low to medium scores (≤ 4) accounted for 11.6% of the total.

### The Difference in Adherence to Labor Analgesia Protocols

As Table 2 shows, the adherence scores were different among the subgroups with different genders, ages, lengths of service, departments, occupations, professional titles, and attitudes. Female medical staff aged over 48 years or with more than 30 years of working experience had a higher rate of adherence. In addition, midwives and obstetricians with senior professional titles and positive attitudes had better labor analgesia adherence. More details are shown in Table 2.

**Table 2:** Descriptive statistics.

**Variables**	***N***	**The scores of adherence of labor analgesia** ***N*** **(%)**							***F***	***P***
		**0**	**1**	**2**	**3**	**4**	**5**	**6**		
**Gender**									11.549	<0.001
Man	1,994	10 (0.5)	13 (0.7)	35 (1.8)	81 (4.1)	203 (10.2)	340 (17.1)	1,312 (65.8)		
Woman	10,620	29 (0.3)	37 (0.3)	99 (0.9)	271 (2.6)	724 (6.8)	1,732 (16.3)	7,728 (72.8)		
**Age**									3.415	0.002
18–27 years old	2,202	12 (0.5)	5 (0.2)	10 (0.5)	42 (1.9)	191 (8.7)	369 (16.8)	1,573 (71.4)		
28–37 years old	6,138	20 (0.3)	30 (0.5)	72 (1.2)	191 (3.1)	452 (7.4)	1,007 (16.4)	4,366 (71.1)		
38–47 years old	2,985	6 (0.2)	8 (0.3)	42 (1.4)	74 (2.5)	201 (6.7)	511 (17.1)	2,143 (71.8)		
over 48 years old	1,289	1 (0.1)	7 (0.5)	10 (0.8)	45 (3.5)	83 (6.4)	185 (14.4)	958 (74.3)		
**Length of service**									3.082	0.005
0–9 years	6,247	25 (0.4)	26 (0.4)	57 (0.9)	167 (2.7)	487 (7.8)	1,080 (17.3)	4,405 (70.5)		
10–19 years	3,790	9 (0.2)	10 (0.3)	43 (1.1)	108 (2.8)	276 (7.3)	594 (15.7)	2,750 (72.6)		
20–29 years	1,889	4 (0.2)	12 (0.6)	33 (1.7)	54 (2.9)	126 (6.7)	299 (15.8)	1,361 (72.0)		
over 30 years	686	1 (0.1)	2 (0.3)	1 (0.1)	23 (3.4)	38 (5.5)	99 (14.4)	522 (76.1)		
**Department**									39.506	<0.001
Operating room	333	3 (0.9)	5 (1.5)	2 (0.6)	9 (2.7)	57 (17.1)	37 (11.1)	220 (66.1)		
Obstetrics (delivery room)	4,681	5 (0.1)	10 (0.2)	27 (0.6)	86 (1.8)	210 (4.5)	688 (14.7)	3,655 (78.1)		
Obstetrics (non-delivery room)	3,768	15 (0.4)	10 (0.3)	31 (0.8)	77 (2.0)	240 (6.4)	633 (16.8)	2,762 (73.3)		
Anesthesiology	3,671	16 (0.4)	25 (0.7)	71 (1.9)	175 (4.8)	395 (10.8)	682 (18.6)	2,307 (62.8)		
Others	161	0 (0)	0 (0)	3 (1.9)	5 (3.1)	25 (15.5)	32 (19.9)	96 (59.6)		
**Occupation**									15.397	<0.001
Anesthesiologist	3,415	10 (0.3)	22 (0.6)	63 (1.8)	162 (4.7)	347 (10.2)	631 (18.5)	2,180 (63.8)		
Anesthesia nurse	416	8 (1.9)	4 (1.0)	9 (2.2)	17 (4.1)	64 (15.4)	68 (16.3)	246 (59.1)		
Obstetrician	3,619	6 (0.2)	8 (0.2)	19 (0.5)	52 (1.4)	176 (4.9)	613 (16.9)	2,745 (75.8)		
Midwife	3,740	1 (0.0)	8 (0.2)	25 (0.7)	73 (2.0)	156 (4.2)	515 (13.8)	2,962 (79.2)		
Obstetric nurse	1,093	12 (1.1)	3 (0.3)	15 (1.4)	35 (3.2)	106 (9.7)	190 (17.4)	732 (67.0)		
Others	331	2 (0.6)	5 (1.5)	3 (0.9)	13 (3.9)	78 (23.6)	55 (16.6)	175 (52.9)		
**Professional titles**									2.428	0.024
Primary and others	5,962	27 (0.5)	21 (0.4)	46 (0.8)	138 (2.3)	465 (7.8)	1,000 (16.8)	4,265 (71.5)		
Middle	4,290	9 (0.2)	22 (0.5)	63 (1.5)	150 (3.5)	297 (6.9)	681 (15.9)	3,068 (71.5)		
Vice-senior	1,775	2 (0.1)	6 (0.3)	20 (1.1)	47 (2.6)	128 (7.2)	288 (16.2)	1,284 (72.3)		
Senior	587	1 (0.2)	1 (0.2)	5 (0.9)	17 (2.9)	37 (6.3)	103 (17.5)	423 (72.1)		
**Attitude**									184.123	<0.001
Strongly disagree	17	0 (0)	1 (5.9)	1 (5.9)	3 (17.6)	1 (5.9)	3 (17.6)	8 (47.6)		
Disagree	18	0 (0)	1 (5.6)	1 (5.6)	3 (16.7)	3 (16.7)	2 (11.1)	8 (44.4)		
Neutral	342	5 (1.5)	11 (3.2)	17 (5.0)	32 (9.4)	45 (13.2)	77 (22.5)	155 (45.3)		
Agree	2,414	8 (0.3)	16 (0.7)	38 (1.6)	104 (4.3)	268 (11.1)	504 (20.9)	1,476 (61.1)		
Strongly agree	9,823	26 (0.3)	21 (0.2)	77 (0.8)	210 (2.1)	610 (6.2)	1,486 (15.1)	7,393 (75.3)		

### Correlation Analysis Between Labor Analgesia Adherence and Other Factors

As Table 3 illustrates, added incentives and related arrangements were positively correlated with adherence. Correspondingly, department collaboration satisfaction, professional development satisfaction, willingness, and gender were related to adherence and support measures. In addition, a positive attitude was also observed as having a similar correlation with adherence and support measures.

**Table 3:** Correlations of variables.

**Variables**	**1**	**2**	**3**	**4**	**5**	**6**	**7**	**8**	**9**	**10**	**11**
1 adherence	-										
2 Added incentives	0.164**	-									
3 Related arrangement	0.289**	0.564**	-								
4 Department collaboration	0.313**	0.445**	0.690**	-							
5 Professional development	0.343**	0.549**	0.706**	0.738**	-						
6 Willingness	0.273**	0.167**	0.260**	0.280**	0.313**	-					
7 Gender	0.071**	0.038**	0.151**	0.103**	0.101**	0.085**	-				
8 Age	0.010	−0.052**	−0.087**	−0.081**	−0.092**	−0.007	−0.119**	-			
9 Length of service	0.018*	−0.025**	−0.050**	−0.047**	−0.062**	0.004	−0.064**	0.833**	-		
10 Professional titles	0.004	−0.038**	−0.103**	−0.086**	−0.091**	−0.013	−0.126**	0.738**	0.730**	-	
11 Attitude	−0.181**	−0.248**	−0.401**	−0.445**	−0.546**	−0.210**	−0.010	0.006	0.009	−0.021*	-

*p < 0.05; **p < 0.01.

### Multiple Linear Regression Analysis of Labor Analgesia Adherence

The continuous variables were entered into the multiple linear regression model. The results are shown in Table 4. The multiple linear regression analysis showed the association of added incentives and satisfaction with adherence to labor analgesia policies. However, a correlation between the length of service and professional titles was not found in the model.

**Table 4:** Multiple linear regression analysis.

	**Unstandardized**		**Standardized**	**T**	**β^1^(95%CI)**
	**coefficients**		**coefficients**		
	**β^1^**	**S.E**.	**β^2^**		
Constant	2.792***	0.094		29.556	2.607 to 2.977
**Supporting measures**					
Added incentives	0.039***	0.008	0.053	5.141	0.024 to 0.054
Related arrangement	0.018***	0.004	0.061	4.715	0.011 to 0.026
**Satisfaction**					
Department collaboration	0.103***	0.015	0.087	6.724	0.073 to 0.133
Professional development	0.069***	0.005	0.203	15.202	0.060 to 0.077
Willingness	0.716***	0.037	0.168	19.390	0.644 to 0.788
**Confounding factors**					
Age	0.007***	0.002	0.067	3.082	0.003 to 0.012
Length of service	−0.039***	0.020	−0.038	−1.942	−0.079 to 0.001
Professional titles	0.026***	0.014	0.024	1.802	−0.002 to 0.055
Department	−0.071***	0.009	−0.069	−7.951	−0.088 to 0.053

***P < 0.001.

### Mediating Effects of Satisfaction on Labor Analgesia Adherence

The estimates derived from the regression model were used to test the conceptual framework. As shown in Table 5, added incentives and related measures had a significant effect on adherence to labor analgesia protocols. Regarding the mediators, the results showed that professional development, department collaboration, and willingness had significant mediating effects on the effect of support measures on adherence to labor analgesia protocols. A significant relationship between support measures and professional development was observed. The correlation coefficient between added incentives and adherence decreased significantly from 0.121 to 0.025 after professional development entered into the regression model. A similar association was seen in analyses of related arrangements.

**Table 5:** Results of mediating effects.

**IV**	**M**	**DV**	**IV-DV**	**IV-M**	**(IV+M)-DV**	
					**IV**	**M**
			**β** **[95%CI]**			
AI	Professional development	Adherence	0.121*** [0.109 to 0.134]	1.232*** [1.199 to 1.265]	0.025*** [0.004 to 0.047]	0.119*** [0.112 to 0.125]
AI	Willingness	Adherence	0.121*** [0.109 to 0.134]	0.029*** [0.026 to 0.032]	0.090*** [0.078 to 0.102]	1.077*** [1.005 to 1.149]
AI	Department collaboration	Adherence	0.121*** [0.109 to 0.134]	0.277*** [0.268 to 0.287]	0.023*** [0.009 to 0.037]	0.353*** [0.332 to 0.375]
AI	Age	Adherence	0.121*** [0.109 to 0.134]	−0.036*** [−0.047 to 0.024]	0.122*** [0.109 to 0.134]	0.020* [0.001 to 0.038]
AI	Length of service	Adherence	0.121*** [0.109 to 0.134]	−0.018** [−0.031 to 0.006]	0.121*** [0.109 to 0.134]	0.023* [0.005 to 0.040]
AI	Professional titles	Adherence	0.121*** [0.109 to 0.134]	−0.026*** [−0.038 to 0.014]	0.121*** [0.109 to 0.134]	0.011 [−0.008 to 0.029]
AI	Attitude	Adherence	0.121*** [0.109 to 0.134]	0.102*** [0.095 to 0.109]	0.094*** [0.081 to 0.107]	0.268*** [0.237 to 0.300]
RA	Professional development	Adherence	0.086*** [0.071 to 0.114]	0.640*** [0.629 to 0.651]	0.027*** [0.021 to 0.034]	0.091*** [0.084 to 0.099]
RA	Willingness	Adherence	0.086*** [0.071 to 0.114]	0.018*** [0.017 to 0.019]	0.069*** [0.064 to 0.074]	0.905*** [0.833 to 0.977]
RA	Department collaboration	Adherence	0.086*** [0.071 to 0.114]	0.173*** [0.170 to 0.177]	0.041*** [0.034 to 0.048]	0.257*** [0.230 to 0.284]
RA	Age	Adherence	0.086*** [0.071 to 0.114]	−0.024* [−0.029 to 0.019]	0.087*** [0.082 to 0.092]	0.038*** [0.020 to 0.056]
RA	Length of service	Adherence	0.086*** [0.071 to 0.114]	−0.014** [−0.019 to 0.009]	0.088*** [0.081 to 0.091]	0.033*** [0.016 to 0.050]
RA	Professional titles	Adherence	0.086*** [0.071 to 0.114]	−0.028** [−0.033 to 0.024]	0.087*** [0.082 to 0.092]	0.036*** [0.018 to 0.054]

β, unstandardized coefficient; IV, independent variable; M, mediator; DV, dependent variable; AI, added incentive; RA, related arrangement. *P < 0.05; **P < 0.01; ***P < 0.001.

Similarly, department collaboration and willingness mediated the effect of support measures on adherence. The correlation coefficient between added incentives and adherence decreased from 0.121 to 0.090 when willingness was entered into the regression model. In addition, age and length of service positively mediated the relationship between support measures and adherence. A partial mediating effect of professional titles on the relationship between added incentives and adherence was observed.

### Moderating Effects of Satisfaction on Labor Analgesia Adherence

As shown in Table 6, Model 1 and Model 2 were developed to estimate the associations among added incentives, professional titles, and the interaction between added incentives and professional titles on adherence. A positive correlation between added incentives and professional titles was observed. The correlation coefficient between added incentives and adherence decreased slightly from 0.121 to 0.109 when professional titles and the interaction between added incentives and professional titles were entered into the regression model. As a result, professional titles moderated the relationship between added incentives and adherence. Likewise, department collaboration moderated the relationship between related arrangements and adherence in Model 3 and Model 4. Attitude had a similar moderating effect in Model 5.

**Table 6:** Results of moderating effects.

**Variable**	**M1**	**M2**	**M3**	**M4**	**M5**	**M6**
	**β** **[95%CI]**					
Added incentive	0.121*** [0.109 to 0.134]	0.109*** [0.008 to 0.139]				
Related arrangement			0.041*** [0.034 to 0.048]	0.120*** [0.097 to 0.143]	0.086*** [0.071 to 0.114]	0.172*** [0.129 to 0.216]
Professional titles	0.011 [0.008 to 0.029]	0.007 [0.005 to 0.013]				
Department collaboration			0.257 [0.230 to 0.284]	0.540*** [0.456 to 0.623]		
Attitude					0.140*** [0.107 to 0.172]	0.421*** [0.291 to 0.550]
**Interaction**						
Added incentive*professional titles		−0.015** [−0.074 to 0.044]				
Related arrangement*department collaboration				0.019*** [0.013 to 0.024]		
Related arrangement*attitude						0.020*** [0.011 to 0.029]

β, unstandardized coefficient. *P < 0.05; **P < 0.01; ***P < 0.001.

## Discussion

Standardized labor analgesia has been recognized as an effective method of promoting safe delivery for pregnant women (48). This study investigated the current situation and associated factors with adherence to labor analgesia protocols by Chinese medical personnel and examined the mediating and moderating effects on satisfaction with adherence. These results are in agreement with a recent study indicating that incentives and related arrangements lead to the improvement of adherence to labor analgesia protocols (49).

The policy, organization, and workforce of labor analgesia need to be further strengthened. Evidence indicates that although the Chinese government strongly supports the development of childbirth analgesia at the national level, it is still in the early stage of implementation, and time is required for further coverage to be developed (18). Based on the medical problems above, it is suggested that the government devote adequate resources and financial support to expand coverage of labor analgesia. In this study, high-scoring medical personnel accounted for 88.1% of the total, but importantly, anesthesiologists and anesthesia nurses had a low level of adherence. A possible explanation is that there is a severe shortage of anesthesiologists (47). There are only 0.5 anesthesiologists per 10,000 people in China, which is far short of the international standard of approximately 2.5 anesthesiologists per 10,000 people (18). However, these trained anesthesiologists are functionally important for improving the level of obstetric anesthesia. The difficulty of obstetric anesthesia and the shortage of staff might contribute to the low adherence to labor analgesia protocols. Thus, medical colleges or associations are suggested to increase anesthesia specialty to expand the scope of anesthesia students and provide more staff training in anesthesiology departments.

Comprehensive incentive measures are conducive to improving satisfaction with labor analgesia in medical personnel. As mentioned above, professional development and willingness played mediating roles in the relationship between supporting measures and adherence (23, 50). This may be explained by the fact that professional development is an important way to develop professional skills and income, which are more attractive to young people with more junior professional titles (40). In addition, comprehensive incentives are likely to improve medical personnel's willingness, which is significantly associated with good knowledge and adherence to labor analgesia standards (39). Comprehensive incentives leading to a sustained improvement in adherence in terms of labor analgesia are expected. These findings suggest that medical institutions should adopt individual incentive measures to improve medical personnel's enthusiasm for the use of labor analgesia in addition to the national-level promotion plan. These insights can help to contribute to the ongoing policy and incentives toward improving adherence to labor analgesia policies, thus addressing pregnant women's needs in the future.

To improve adherence to labor analgesia protocols, capacity-building programmes for medical institutions are recommended to enhance department collaboration and change medical personnel's attitudes. The level of departmental collaboration is an essential way to strengthen doctors' medical skills, which suggests the significance of cooperation between medical teams for treatment (43, 51). In addition, medical personnel's attitudes toward labor analgesia deserve more attention. It was found that the attitude of professionals is significantly related to labor analgesia. Some medical personnel believe that labor analgesia affects pregnant women's health and thus reduces their use of labor analgesia (39). Increasing the risk of illness and delaying delivery are common reasons why medical personnel are unwilling to provide labor analgesia (52). However, the National Association of Obstetricians and Gynecologists (ACOG) passed a series of clinical trials and proved that labor analgesia is unrelated to the incremental maternal prevalence (53, 54). This misunderstanding may be the cause of low adherence (55). Therefore, strengthening training for the target group and enhancing interdepartmental cooperation are suggested for improving low adherence among certain groups, such as anesthesiologists and anesthesia nurses. Meanwhile, labor analgesia in China is currently concentrated mainly in specialty hospitals. It is recommended that general hospitals improve their ability to provide labor analgesia services by preparing specific internal protocols and arranging adequate training for the involved personnel.

## Limitation

Some limitations of this study need to be mentioned. First, this study used a cross-sectional survey. It therefore could not determine the long-term status of the adherence to labor analgesia protocols in Chinese medical personnel. A cross-sectional study has limited ability to infer causality and the potential for reverse causation. In addition, retrospective self-assessment may lead to bias, and medical personnel may miss some specific details during the investigation process. In addition, this survey was conducted through a web-based questionnaire, and the quality of the investigation process was difficult to control.

## Conclusion

This study aimed to investigate the current situation and associated factors with the adherence to labor analgesia protocols by Chinese medical personnel. The results show that willingness had direct and indirect beneficial effects on adherence to labor analgesia protocols. Satisfaction played an important role in the improvement of labor analgesia. The government should continue to reinforce the promotion of labor analgesia to continue to improve the proportion of financial support. More training opportunities should be provided for more anesthesiologists and anesthesia nurses to enhance knowledge about labor analgesia knowledge. More capacity-building programmes should be launched to improve interdepartmental collaboration. An effective incentive mechanism needs to be designed to attract more medical staff, especially skilled personnel, to participate in labor analgesia work.

## Data Availability Statement

The raw data supporting the conclusions of this article will be made available by the authors, without undue reservation.

## Ethics Statement

Written informed consent was obtained from the individual(s) for the publication of any potentially identifiable images or data included in this article.

## Author Contributions

DL: analyzed the data and wrote the original draft preparation. CLo, SL, YX, FC, RZ, and ST: gave the critical feedback. All authors: conceptualization and data collection. All authors contributed to the article and approved the submitted version.

## Conflict of Interest

The authors declare that the research was conducted in the absence of any commercial or financial relationships that could be construed as a potential conflict of interest.

## Publisher's Note

All claims expressed in this article are solely those of the authors and do not necessarily represent those of their affiliated organizations, or those of the publisher, the editors and the reviewers. Any product that may be evaluated in this article, or claim that may be made by its manufacturer, is not guaranteed or endorsed by the publisher.
